# The Proteomics Standards Initiative Standardized Formats for Spectral Libraries and Fragment Ion Peak Annotations: mzSpecLib and mzPAF

**DOI:** 10.1021/acs.analchem.4c04091

**Published:** 2024-11-08

**Authors:** Joshua Klein, Henry Lam, Tytus D. Mak, Wout Bittremieux, Yasset Perez-Riverol, Ralf Gabriels, Jim Shofstahl, Helge Hecht, Pierre-Alain Binz, Shin Kawano, Tim Van Den Bossche, Jeremy Carver, Benjamin A. Neely, Luis Mendoza, Tomi Suomi, Tine Claeys, Thomas Payne, Douwe Schulte, Zhi Sun, Nils Hoffmann, Yunping Zhu, Steffen Neumann, Andrew R. Jones, Nuno Bandeira, Juan Antonio Vizcaíno, Eric W. Deutsch

**Affiliations:** † Program for Bioinformatics, Boston University, Boston, Massachusetts 02215, United States; ‡ Department of Chemical and Biological Engineering, The Hong Kong University of Science and Technology, Clear Water Bay, 999077 Hong Kong, P. R. China; § Mass Spectrometry Data Center, National Institute of Standards and Technology, 100 Bureau Drive, Gaithersburg, Maryland 20899, United States; ∥ Department of Computer Science, University of Antwerp, 2020 Antwerpen, Belgium; ⊥ European Molecular Biology Laboratory, European Bioinformatics Institute (EMBL-EBI), Wellcome Trust Genome Campus, Hinxton, Cambridge CB10 1SD, United Kingdom; # VIB-UGent Center for Medical Biotechnology, VIB, 9052 Ghent, Belgium; 7 Department of Biomolecular Medicine, Faculty of Medicine and Health Sciences, Ghent University, 9052 Ghent, Belgium; 8 Thermo Fisher Scientific, 355 River Oaks Parkway, San Jose, California 95134, United States; 9 RECETOX, Faculty of Science, Masaryk University, Kotlářská 2, 60200 Brno, Czech Republic; 10 Lausanne University Hospital, CH-1011 Lausanne, Switzerland; 11 Database Center for Life Science, Joint Support Center for Data Science Research, Research Organization of Information and Systems, Chiba 277-0871, Japan; 12 School of Frontier Engineering, Kitasato University, Sagamihara 252-0373, Japan; 13 Center for Computational Mass Spectrometry, Department of Computer Science and Engineering, University of California, San Diego, California 92093-0404, United States; 14 National Institute of Standards and Technology (NIST) Charleston, Charleston, South Carolina 29412, United States; 15 Institute for Systems Biology, Seattle, Washington 98109, United States; 16 Turku Bioscience Centre, University of Turku and Åbo Akademi University, FI-20520 Turku, Finland; 17 Biomolecular Mass Spectrometry and Proteomics, Bijvoet Center for Biomolecular Research and Utrecht Institute of Pharmaceutical Sciences, Utrecht University, Padualaan 8, 3584, CH, Utrecht, The Netherlands; 18 Institute for Bio- and Geosciences (IBG-5), Forschungszentrum Jülich GmbH, 52428 Jülich, Germany; 19 National Center for Protein Sciences (Beijing), Beijing Institute of Lifeomics, #38, Life Science Park, Changping District, Beijing 102206, China; 20 Computational Plant Biochemistry, Leibniz Institute of Plant Biochemistry, 06120 Halle, Germany; 21 German Centre for Integrative Biodiversity Research (iDiv), Halle-Jena-Leipzig, 04103 Leipzig, Germany; 22 Institute of Systems, Molecular and Integrative Biology, University of Liverpool, Liverpool L69 3BX, United Kingdom; 23 Skaggs School of Pharmacy and Pharmaceutical Sciences, University of California San Diego, La Jolla, California 92093, United States

## Abstract

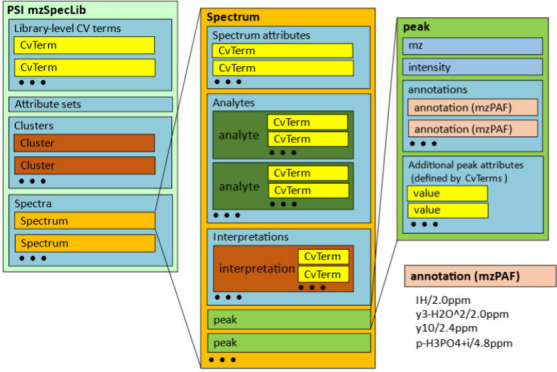

Mass spectral libraries are collections of reference spectra, usually associated with specific analytes from which the spectra were generated, that are used for further downstream analysis of new spectra. There are many different formats used for encoding spectral libraries, but none have undergone a standardization process to ensure broad applicability to many applications. As part of the Human Proteome Organization Proteomics Standards Initiative (PSI), we have developed a standardized format for encoding spectral libraries, called mzSpecLib (https://psidev.info/mzSpecLib). It is primarily a data model that flexibly encodes metadata about the library entries using the extensible PSI-MS controlled vocabulary and can be encoded in and converted between different serialization formats. We have also developed a standardized data model and serialization for fragment ion peak annotations, called mzPAF (https://psidev.info/mzPAF). It is defined as a separate standard, since it may be used for other applications besides spectral libraries. The mzSpecLib and mzPAF standards are compatible with existing PSI standards such as ProForma 2.0 and the Universal Spectrum Identifier. The mzSpecLib and mzPAF standards have been primarily defined for peptides in proteomics applications with basic small molecule support. They could be extended in the future to other fields that need to encode spectral libraries for nonpeptidic analytes.

## Introduction

Mass spectral libraries (or “spectral libraries” for short hereafter) are collections of fragment ion mass spectra and their originating analytes (if known) that are intended to be used as a reference for future spectral analysis. An MS2 spectrum records the characteristic fragmentation patterns of an analyte, in terms of mass-to-charge ratios and abundances of fragment ions, that reproducibly occur when the analyte is fragmented in the mass spectrometer by one of various methods. Because of this, mass spectral libraries have important applications in analytical chemistry, particularly in the fields of proteomics and metabolomics/lipidomics.^1−4^

In proteomics, spectral libraries are generally compiled from a large amount of data acquired on complex protein mixtures.^5^ Often the data were acquired to answer various biological questions as a primary goal rather than to generate spectral libraries. In metabolomics and lipidomics, spectral libraries are more often generated by injecting pure analytes onto the mass spectrometer, thereby conclusively linking the MS2 spectrum and the analyte.^6^ This requires the dedicated effort by library builders, most notably the National Institute of Standards and Technology (NIST) of the United States Government, some academic groups (e.g., METLIN spectral libraries)^7^ and vendors of mass spectrometers.

Especially in proteomics, applications also exist for libraries that contain unidentified MS2 spectra (exclusively or not). Such spectral libraries are sometimes referred to as spectral archives.^8^ For spectral archives to be useful, spectra are often grouped by spectral similarity into clusters by some clustering algorithm. It is often, but not always, assumed that spectra in a cluster originate from the same analyte or from highly related analytes, on account of their spectral similarity. It should also be noted that in recent years, due to the advances in artificial intelligence, it has become possible to predict the characteristic fragmentation patterns of the analytes of interest, for both peptides and small molecules.^9,10^ Such predicted spectra can also be compiled into *in silico* spectral libraries and utilized in a way similar to traditional spectral libraries built from real data.

In proteomics, although sequence database searching is still the method of choice in data-dependent acquisition (DDA) experiments, spectral library searching offers notable advantages.^11^ First, they enable faster identification due to a smaller search space of observable peptides. Second, these libraries can increase the specificity and sensitivity of peptide identification tools by using known fragment ion intensities rather than solely relying on the calculated mass-to-charge ratios of the expected fragment ions. On the other hand, in data-independent acquisition (DIA) experiments, where the fragmentation of multiple precursors simultaneously leads to convoluted and hard-to-interpret spectra, spectral libraries play a crucial role in data analysis, in particular in peptide-centric approaches that seek to monitor anticipated fragment ions coeluting over time.^12^ Similarly, practitioners of targeted quantitative workflows such as selected reaction monitoring (SRM) depend on spectral libraries to design MS-based “assays” prior to the experiment, so that the mass spectrometer can be instructed to isolate and quantify specific fragment ions.^13^

Peak annotations are useful information for many applications of spectral libraries. Annotation refers to the assignment of observed peaks in a spectrum to the fragment ions responsible for them and can be obtained manually or computationally. The peak annotations (or lack thereof) provide the best indication of whether the observed spectrum is of high quality and can be adequately explained by analyte identification. For example, a spectrum with a large number of unannotated peaks is a telltale sign that the spectrum may be incorrectly identified or is contaminated with another analyte. Some peaks in a spectrum, such as those resulting from cleavages of isobaric labeling reagents for quantifications, have special uses and should be clearly marked. Additionally, spectral library search engines can assign different weights to the matching of fragment ion peaks of different types due to their different information content.^5,14^ However, since each spectrum may have hundreds of peaks, peak annotations can account for a large fraction of the storage space and parsing time of a spectral library. Therefore, encoding peak annotations compactly but unambiguously is a highly desirable feature of any spectral library format.

Regardless of the source of the libraries and their intended applications, there is a clear need for various metadata associated with the library and its constituent spectra. There have been for many years several different popular spectral library formats in proteomics, including the MSP format used by NIST, the splib format used by SpectraST,^5,15^ the blib format used by Bibliospec,^16^ simple yet *ad hoc* tab-separated formats for DIA applications, and others. Similarly, in metabolomics, the MSP format and the Mascot Generic Format (MGF) are commonly used. While these formats are fit for the purpose of storing the spectra themselves, they all lack an adequate and well-documented mechanism for providing rich metadata about the spectra, including their provenance. Several of these formats store limited metadata using *ad hoc* keywords in a comment field. There are common conventions, but these have evolved over the years with no formal documentation or consensus among tools or domains. As spectral libraries become ever more important for mature and emerging applications, a need for a community standard spectral library format with extensive metadata has been identified.^17^ It is recognized that a standardized format for spectral libraries will greatly facilitate the systematic compilation, dissemination, and utilization of spectral libraries in the research community.

The Human Proteome Organization (HUPO) Proteomics Standards Initiative (PSI) has developed multiple open community data standards (e.g., mzML, mzidentML, mzTab), guidelines, and controlled vocabularies/ontologies, among other outputs, since its inception in 2002.^18−20^ Here we describe the HUPO PSI mzSpecLib and mzPAF standards designed to encode spectral libraries and annotations of peptide fragment ion peaks, respectively. They have been split into separate standards since fragment ion peaks may be annotated in contexts outside of spectral libraries, such as spectrum display software. However, they are described here together since they will most often be jointly used. We first provide an overview of the mzSpecLib format and then the mzPAF format followed by descriptions of current software implementations that may guide further implementations.

## Methods

The development of mzSpecLib and mzPAF started in 2019. Since then, it has been an open process via conference calls, in addition to discussions at the annual PSI meetings and smaller workshops. Both technical specification documents were submitted to the PSI Document Process^21^ for review, during which time external anonymous reviewers not involved in the development process provided their feedback. The document was also made available for comments by the public, enabling broad input on the specifications. The latest versions of the specification documents, up-to-date information on software implementations, and information on future versions of mzSpecLib and mzPAF are available at https://psidev.info/mzSpecLib and https://psidev.info/mzPAF, respectively.

Historically the PSI has developed open and standardized data formats mostly for proteomics applications that have focused on storing mass spectra as generated by an instrument (the mzML format),^22^ and formats that have focused on storing the downstream identifications of those generated mass spectra (the mzIdentML^23,24^ and mzTab^25^ formats). These formats have been kept separate intentionally, with the identification formats designed to refer to the spectrum-containing format. This was done primarily to control file sizes since the serialization of spectra takes substantial space and replicating the spectra in the identification format seemed inefficient.

Spectral libraries are designed with an opposite approach: to unite the spectra and their interpretations into a single format. The significant difference, however, is that spectral libraries are intended to contain single or aggregated spectra that are deemed important, collated across potentially many experiments, to form a reference set of spectra that may be used for downstream processing. Spectral libraries are, however, not intended to capture the complex output of mass spectrum processing pipelines (for which mzIdentML is designed).

During the development process, the working group identified the following design principles for the mzSpecLib format. First, it was decided that the standard should take the form of a common data model with multiple interconvertible serializations that can serve different needs. For example, if human readability is desired, a text-based serialization would be the most suitable, while a JSON file would be best for software-application data exchange. These two serializations are implemented at the present stage, but more can be added in the future. This design choice is unique among PSI formats but is necessary to reduce the barrier of entry and to address the sometimes-conflicting demands of library builders and users. We expect that applications that demand efficiency will need to develop their own serialization and indexes since it is difficult for all needs to be anticipated. For example, a serialization that optimizes for random access may not be optimal for storing and reading data for machine learning applications. Defining mzSpecLib to be a data model rather than a file format is an important first step in ensuring that different serializations developed by anyone can be interconverted.

Second, all metadata are defined via terms from a controlled vocabulary (CV), primarily the PSI-MS,^26^ which is actively maintained by this PSI working group. This forces the producers and consumers of spectral libraries to use mutually agreed terminology with clear definitions to refer to the same concept. For concepts that are outside the scope of the PSI-MS, e.g., sample metadata terms, it is permissible to use compact URIs (CURIEs) (https://www.w3.org/TR/2010/NOTE-curie-20101216/) from other CVs, e.g., EFO:0000434|ecotype = EFO:0005148|Col-0 from the Experimental Factor Ontology (EFO),^27^ with prefixes from bioregistry.io.^28^ In addition, to ensure flexibility and extensibility, there was no formal restriction in the specification as to what terms may or may not be used in a spectral library as long as they are defined in the CV. However, a table of commonly used terms is provided to guide the adoption of best practices in a separate “living” document maintained online as an addendum to the format. Third, the format should provide a mechanism to minimize redundancy and file size by not encoding repetitive metadata in every spectrum. Meanwhile, any mechanism to do so must also ensure that the data integrity of the library can be maintained as libraries are split or merged and as library entries are added or deleted. Finally, to set a manageable scope for the project, the inaugural version of the format will primarily support proteomics applications with basic small molecule support. Nevertheless, care has been taken so that the overall design can be extended to other MS fields in the future (metabolomics, lipidomics, glycomics, etc.).

For mzPAF, the working group identified the following design principles for the encoding of peak annotations. First, mzPAF should be designed as a compact, human-readable, and unambiguous software-parsable format. Second, the format is also mapped to an object model that may be serialized as a JSON for certain applications. Third, to set a manageable scope for the project, mzPAF is designed for linear peptides with simple modifications, i.e., those routinely identified by typical proteomics pipelines, and for fragmentation methods commonly used in proteomics such as collision-induced dissociation (CID), higher-energy collisional dissociation (HCD), and electron-transfer dissociation (ETD). Finally, as in the case of mzSpecLib, although there are some provisions for annotating small molecules, it is expected that for other major classes of analytes (small molecules, glycans, lipids, glycopeptides, cross-linked peptides, etc.), extended peak annotation formats should be defined in the future, ideally compatible with this format.

## Results and Discussion

### mzSpecLib Format

The mzSpecLib specification primarily takes the form of a data model with multiple possible serializations of that data model. The specification describes two serializations: text-based serialization and JavaScript Object Notation (JSON) serialization. Other, potentially more space-efficient serializations for additional use cases are envisioned and are in progress. Lossless interconversion between these serialization methods is provided by a reference implementation Python library (https://github.com/HUPO-PSI/mzspeclib-py), and other implementations are welcome and forthcoming.

**Figure 1:**
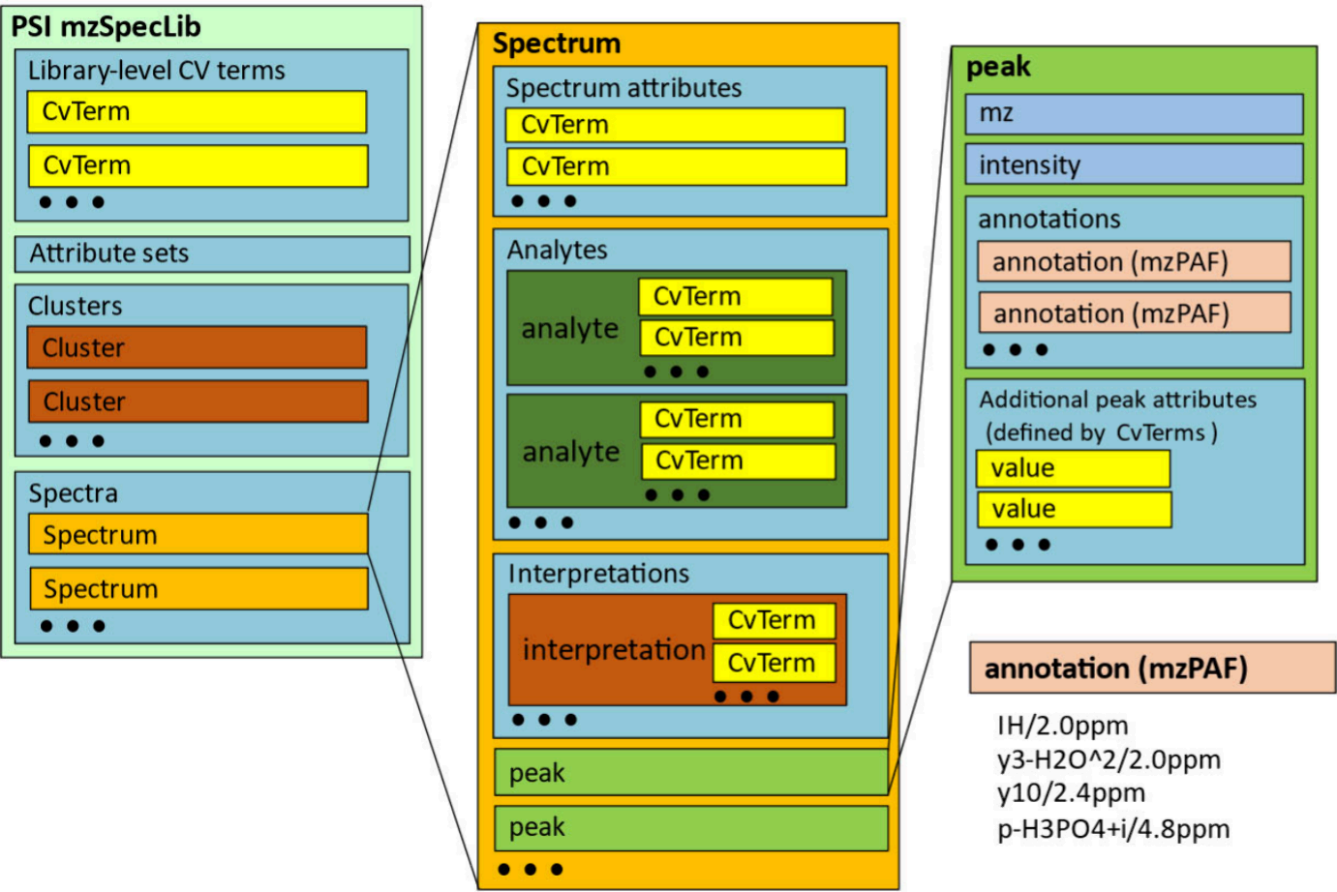
Overview of the mzSpecLib data model. The model consists of six main components: the library metadata, clusters, spectra, analytes, peaks, and the peak annotations in mzPAF.

The data model consists of six main components (Figure 1). The top level contains basic metadata that describe the library itself. At this level, attribute sets can be defined for entry-level metadata that are shared by many library entries. Optionally, a list of spectrum clusters follows, which consists of references (links) to the library entries belonging to each spectrum cluster. This cluster feature is intended to capture the output of spectrum clustering algorithms,^29−33^ which collect sets of similar spectra (“clusters”) thought to be derived from the same or similar precursor ions, even when the identity of not all precursor ions may be known.

The next component describes each individual spectrum entry, both the metadata about each spectrum and a list of peaks. Spectrum metadata describes attributes inherent to the spectrum and is accompanied by metadata that describe the one or more putative analytes that produced it and metadata describing the interpretations that combine one or more of the putative analytes. The spectra themselves may be individual observed spectra, consensus spectra derived from multiple individual input spectra, or predicted spectra based on one of the many available algorithms. Most libraries comprise collections of consensus spectra that are derived from DDA data, but libraries may also be derived from DIA data, in which fragment ion peaks are selected based on having an ion trace that is compatible in profile (i.e., maximum retention time and shape) with other member fragment ion peaks and/or being a predicted fragment of the putative analyte.

The analytes are precursor ions (typically charged peptidoforms in this initial implementation but potentially extensible to other kinds of charged molecules). The interpretations are the groups of analytes that explain the spectrum. In the simplest and most common case, a single interpretation includes one analyte. However, the format supports additional cases such as the definition of analytes 1, 2, and 3, with two possible interpretations for the spectrum, one of which is a blend of analytes 1 + 2 and the other is a blend of analytes 2 + 3.

The next component describes each peak, including the *m*/*z* values, intensities, and aggregation metadata (for when a spectrum is an aggregated spectrum and aggregation metrics are available).

The final component describes one or more proposed annotations for each peak. This information is encoded in the mzPAF peak annotation standard, as described below and found at http://psidev.info/mzPAF.

The text serialization of mzSpecLib is somewhat similar to the popular NIST MSP format but is formally standardized, which MSP never was. Furthermore, it adds important features such as support for spectral clustering output, support for unidentified (but commonly observed) spectra, support for multiple analytes per spectrum, and alternative hypotheses for the interpretation of each spectrum. It also explicitly makes use of the PSI-MS controlled vocabulary, rather than using somewhat *ad hoc* keywords in the comment field that were never properly defined. Thus, while the formal mzSpecLib specification is expected to remain fixed for a long time, its use can be sufficiently flexible via the externally maintained controlled vocabulary. This principle is analogous to that followed in all PSI data standards.

Each of the aspects mentioned above are documented in extensive detail—often with a dedicated subsection to each aspect—in the formal mzSpecLib specification available at https://psidev.info/mzspeclib. Implementers and interested readers are referred to the full specification document for further details.

**Figure 2:**
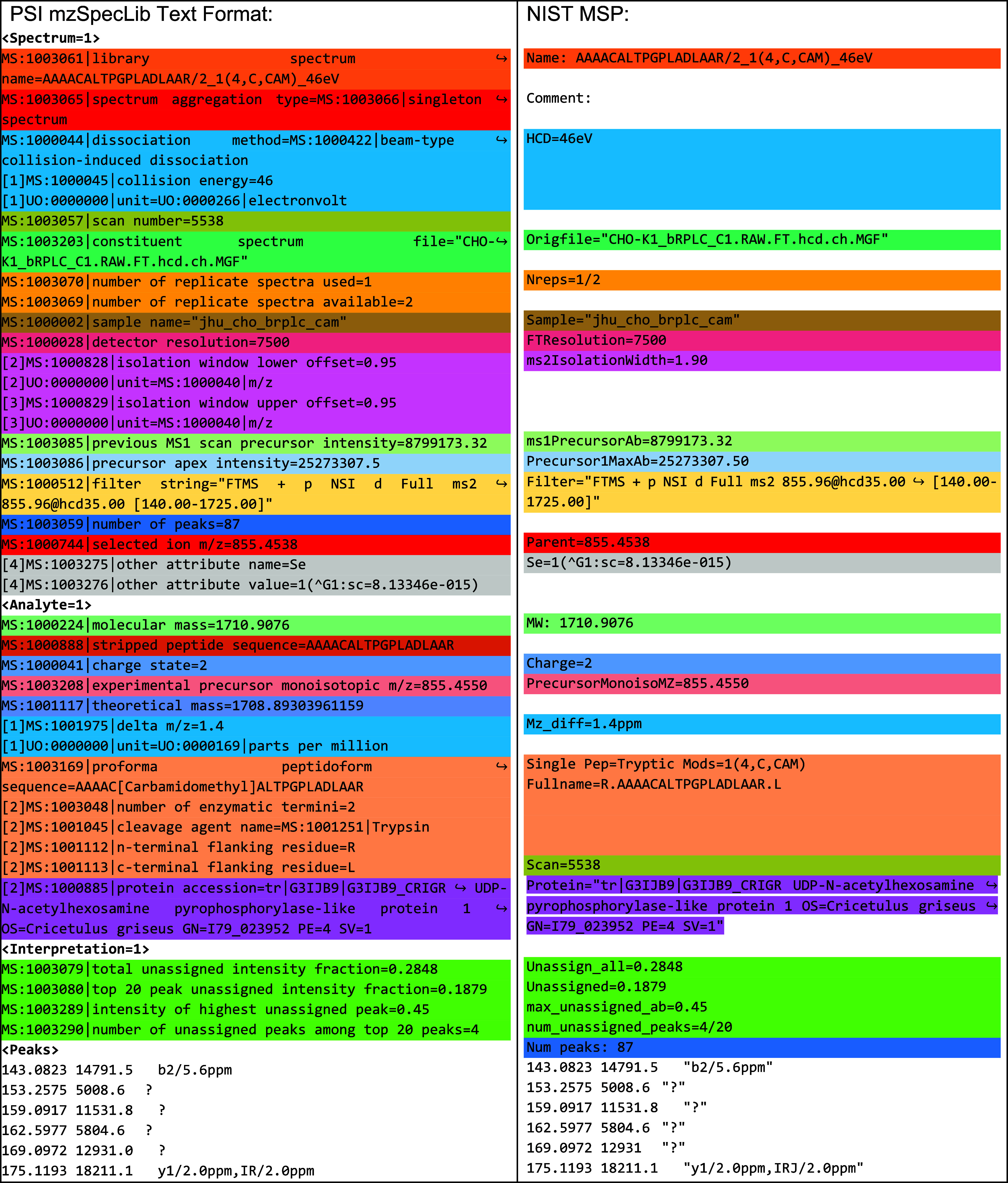
A side-by-side comparison of a spectrum from NIST’s chinese_hamster_hcd spectral library (https://chemdata.nist.gov/dokuwiki/doku.php?id=peptidew:lib:cho_20180223) in MSP format and the equivalent spectrum in mzSpecLib’s text encoding. Note that in the MSP file all of the lines between “Comment” and “Num Peaks” are key-value pairs within a single Comment line but separated out here for better readability. The MSP spectrum comments are color-mapped to their equivalent controlled vocabulary terms. Wide lines that are wrapped for display purposes are marked with a wrapped arrow symbol.

**Figure 3:**
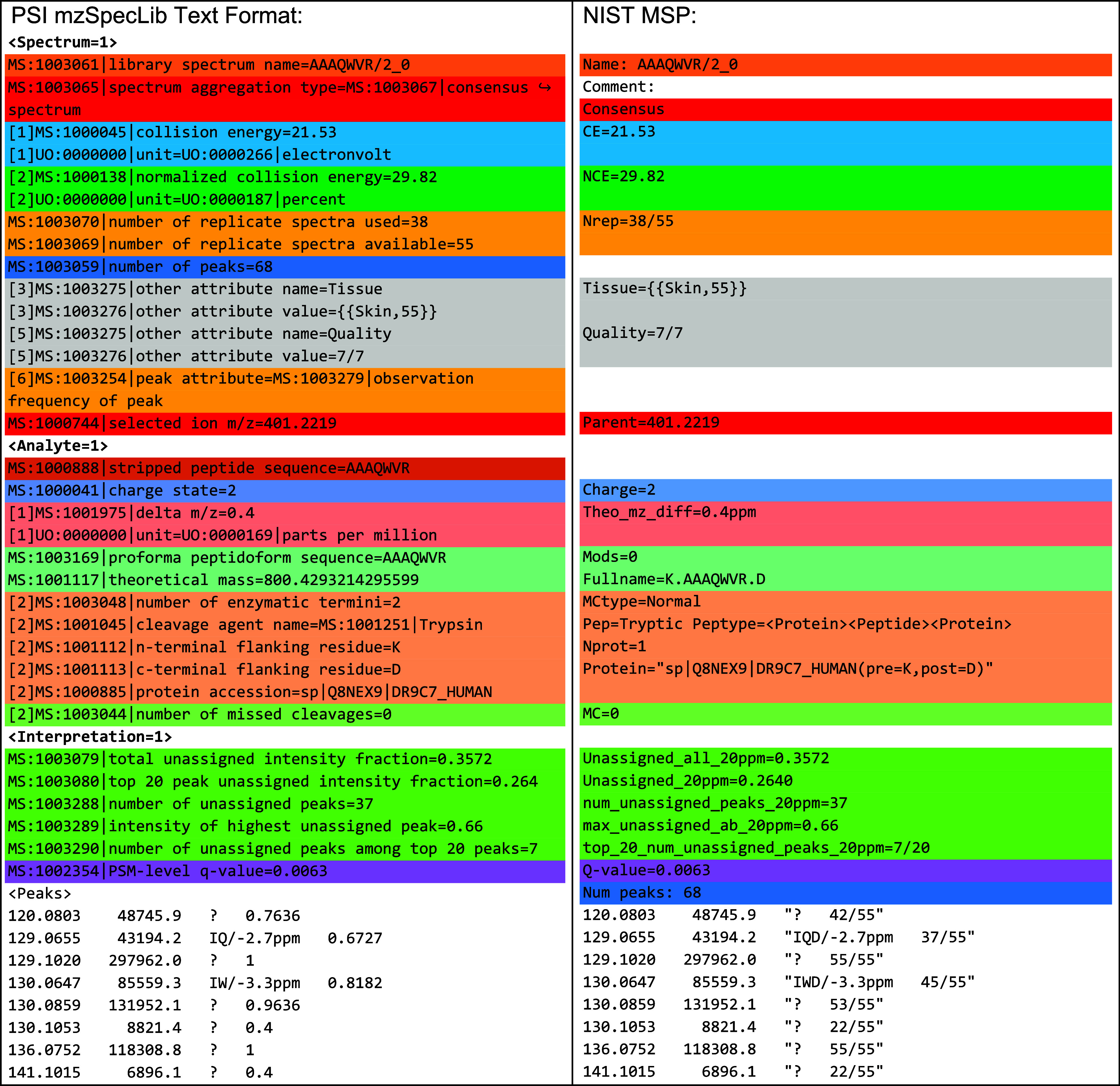
A side-by-side comparison of a spectrum from NIST IARPA3_best_tissue_add_info spectral library (https://chemdata.nist.gov/dokuwiki/doku.php?id=peptidew:lib:human_skin_hair) in MSP format and the equivalent spectrum in mzSpecLib’s text encoding. See https://github.com/HUPO-PSI/mzSpecLib/blob/master/examples/NIST/IARPA3_best_tissue_add_info.head.mzlib.txt for a more complete example. Wide lines that are wrapped for display purposes are marked with a wrapped arrow symbol.

In Figure 2 and Figure 3, we show spectra from different NIST spectral libraries and their equivalent mzSpecLib text renderings, highlighting the mapping between the MSP key-value pairs and the controlled vocabulary terms. By cataloging many MSP files from NIST and other sources, we identified synonymous or conditionally equivalent keys. For example, at least eight distinct term keys were found that corresponded to the analyte precursor *m*/*z*, six terms for scan polarity, and eight terms for collision energy (CE). For example, in Figure 2 the HCD key is equivalent to the CE key in Figure 3.

In some cases, a single MSP comment implies multiple attributes, as in the case of “Nreps” mapping to “MS:1003070|number of replicate spectra used” (accession of the CV term in the PSI-MS controlled vocabulary and its name) and “MS:1003069|number of replicate spectra available”, and implying that peak aggregation of type “MS:1003254|peak attribute = MS:1003279|observation frequency of peak” should be present but may not be available.

mzSpecLib is more precise with respect to concepts that may seem interchangeable in other contexts. For example, the *m*/*z* of the selected ion (MS:1000744|selected ion *m*/*z*), the theoretical *m*/*z* of the annotated analyte (MS:1003053|theoretical monoisotopic *m*/*z*), and the experimentally determined monoisotopic peak *m*/*z* (MS:1003208|experimental precursor monoisotopic *m*/*z*) for the analyte or selected ion might all be called the precursor *m*/*z*, but they mean different things. By having explicit contexts, mzSpecLib makes it clear which piece is the subject of each attribute, especially when there are multiple interpretations or multiple analytes per spectrum. The specification also provides recommendations for using standardized formats for defining analytes, using ProForma 2.0^34^ for peptides, and either SMILES or InChI for small molecules. The ProForma 2.0 specification allows the description of quite complex post-translational modifications and other artifactual mass modifications, including modification localization ambiguities.

**Figure 4:**
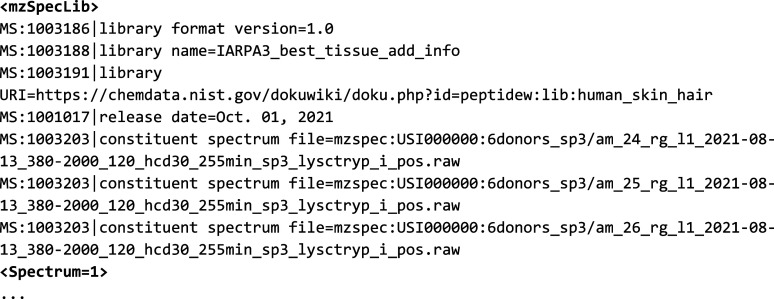
mzSpecLib library file header snippet taken from the conversion of the NIST IARPA3_best_tissue_add_info spectral library (https://chemdata.nist.gov/dokuwiki/doku.php?id=peptidew:lib:human_skin_hair) to mzSpecLib text format. This header was produced by incorporating the metadata from an external text file (https://chemdata.nist.gov/download/peptide_library/libraries/skin_hair/IARPA3_all.out.zip) because the MSP format had no suitable representation for the relevant details. See https://github.com/HUPO-PSI/mzSpecLib/blob/master/examples/NIST/IARPA3_best_tissue_add_info.head.mzlib.txt for a more complete example.

The mzSpecLib text format uses sections to explicitly organize information into different contexts, using “<”, the section opening expression, followed by “>” to denote a new section. The format begins with a header section, “<mzSpecLib>”, containing library-level metadata, often missing in plain-text formats, describing attributes like where the library came from or was made, its identifiers, and license if that information is available (Figure 4). This header may include information about which raw mass spectrometry data files were included in the library using the Universal Spectrum Identifier (USI)^35^ notation for data files. It is not currently standard practice in the field to provide the false discovery rate (FDR) of a spectrum library, although every library probably has at least some false positives. It is encouraged that producers of mzSpecLib libraries provide a global FDR of a library in the header via a term such as MS:1002350|PSM-level global FDR = 0.nnn when it is known. Furthermore, the specification allows the writer to describe the procedure and software tools used to generate FDR (or *q*-value) estimates, e.g., by including terms such as MS:1001456|analysis software. The library header may also declare sets of shared attributes to reduce repetition in sections below it, in the style of the cascading configuration.

Next, an mzSpecLib library can list any number of “<Cluster = ${key}>” entries, describing related groups of spectra inside or outside the library, followed by any number of “<Spectrum = ${key}>” entries, describing library spectra including any acquisition or generation attributes, interpretations as sets of zero or more analytes, and a peak list that may include peak annotation or aggregation statistics. All of these entities are defined either in terms of controlled vocabulary attributes or via an externally defined grammar-data model pair permitted by the specification.

### mzPAF Format

In the mzSpecLib format, it is possible to annotate individual peaks, as is already done in spectral libraries from NIST (https://chemdata.nist.gov/dokuwiki/doku.php?id=peptidew:start), SpectraST,^5^ and PeptideAtlas.^36^ However, there have been several different styles of annotation in the past (even from a single provider). To address this, mzPAF describes a single common peak annotation format standard for peptides that is recommended for all peptide libraries and related applications for which peak annotations are desirable.

The mzPAF specification is heavily based on the formatting used in the NIST MSP format and the SpectraST sptxt format. These precursor formats were quite similar but not exactly the same and were never fully documented. NIST MSP annotations have undergone small changes over the years. Those annotation formats are based on the original nomenclature proposals published by Roepstorff and Fohlman,^37^ which was further refined by Biemann.^38^ Participation from NIST and SpectraST developers and many others has led to the development of this unified standard.

The mzPAF format, as described in the Methods, is designed for linear peptides with simple modifications, i.e., those routinely identified by typical proteomics pipelines (with or without enrichment methods), and for fragmentation methods commonly used in proteomics. However, there are some provisions for annotating small molecules (e.g., contaminants in a predominantly peptide spectrum) as well as unusual fragments.

**Figure 5:**
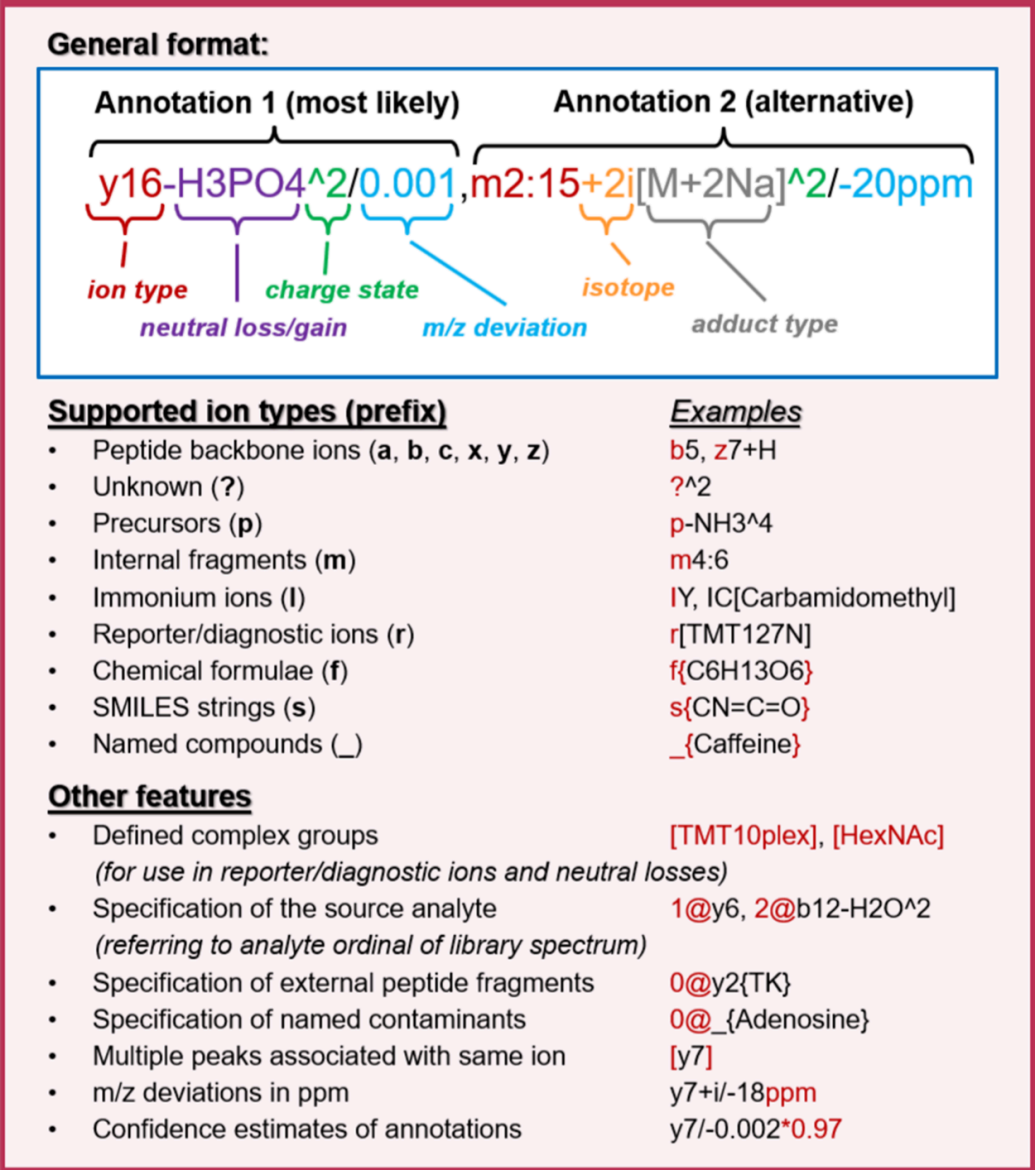
Main features of the mzPAF format. An example peak annotation with common components is shown along with a list of supported ion types (with prefix in parentheses) and other features of the format. Detailed descriptions can be found in the documentation.

Figure 5 highlights the main aspects of mzPAF with an example and a list of major features. At the top are two example annotations, separated by a comma, indicating that one or both are potential interpretations of a peak, with the first deemed most likely. The first annotation describes a doubly charged y16 ion (a chain of 16 residues beginning at the C terminus of a peptide) with an H_3_PO_4_ neutral loss (a common neutral loss for a phosphorylated peptide) somewhere on those 16 residues. The *m*/*z* delta between measured *m*/*z* and predicted *m*/*z* is 0.001. The second annotation shows a second isotope of a doubly charged internal fragmentation ion (“m” for middle) of residues 2 through 15 (counting from the N terminus) that acquired its charge via two sodium ions. The delta of measured *m*/*z* minus computed *m*/*z* for the annotated fragment is −20 ppm.

The central panel of Figure 5 lists the mzPAF-supported ion types, including the customary abcxyz designations, “m” for internal fragmentation ions, “?” for unidentified ions, “p” for precursor-related ions, “I” for immonium ions, “r” for reference ions (such as isobaric labeling related ions), “f” for arbitrary chemical formulas, “s” for SMILES strings, and “_” for named compounds. Examples of each of these ion types are also provided.

The bottom panel of Figure 5 lists some notable features of the standard. Certain CV terms from a list that accompanies the specification or the Unimod CV^39^ for protein modifications may be specified in square brackets. An analyte number may be specified before each annotation (e.g., “1@” or “2@”) for cases where more than one analyte may be present in the spectrum (“0@” indicates an unspecified contaminant ion). Such contaminant ions may take a few different forms, such as “0@y2{TK}” to indicate a y2 type ion from some unspecified ion (ending with TK on the C terminus), or “0@_{Adenine}” to indicate a fragment ion corresponding to a nucleotide fragment perhaps from an RNA molecule that entered the selection window (common in phospho-enriched data sets). If multiple peaks gather the same annotation since they are closer together than the annotation *m*/*z* tolerance, the less likely duplicates may be enclosed in square brackets. The *m*/*z* delta values are always expressed as measured minus computed, with default units as *m*/*z* and deltas in parts per million listed with a suffix of “ppm”. Finally, the confidence of one or more annotations may be specified with a “*” followed by a number between 0 and 1, inclusive (e.g., *0.97). It may be used to indicate the relative confidence between alternative explanations or to indicate a confidence that a proposed explanation is truly the source of measured peak (perhaps based on a model of the *m*/*z* deltas for the spectrum and likelihood that such a fragment ion would be present).

All of these features are described in substantially greater detail—often in a dedicated subsection—in the formal specification document available at https://psidev.info/mzPAF/. Although the compact text notation described in Figure 5 and the text above is the most common serialization for spectral libraries and annotated-spectrum depictions, a formal object model that may be serialized in JSON is also described in the specification and may be useful in software implementations and transfer of information between software implementations.

Not only is the compact mzPAF text format human readable (with some expertise), it is also unambiguously software-parsable, via either a regular expression or a state machine parser. Such parsers are already implemented in the Python mzPAF package (https://github.com/HUPO-PSI/mzpaf), and also for several other languages. It is hoped that the vast majority of software tool writers can use existing parsing code without needing to implement their own parser (which is a nontrivial task). As explained in the Methods, it is expected that for other major classes of analytes extended peak annotation formats should be defined in the future.

### Resources and Implementations

Multiple resources and implementations are already available for mzSpecLib and mzPAF, and it is expected that additional resources and implementations will follow in parallel with the adoption of the format. The reference implementations that have been developed in parallel with the specification, and used to test the specification, are the Python packages mzspeclib and mzpaf, which are available on PyPI and in their GitHub repositories at https://github.com/HUPO-PSI/mzspeclib-py and https://github.com/HUPO-PSI/mzpaf, respectively. These packages provide a complete set of classes and methods needed to read and write both mzSpecLib and mzPAF annotations. Furthermore, the mzspeclib package provides a spectral library data conversion tool that is able to convert various other formats (such as the NIST MSP format) into mzSpecLib files and the reverse as well for a subset of formats. This allows most existing libraries to be converted to mzSpecLib, and mzSpecLib libraries to be converted to another format, for example, in order to be used with a software package that does not yet support mzSpecLib natively.

In addition to the specification documents and related reference files, there are example files in the GitHub repositories listed above. The examples at the mzPAF site provide a series of example spectra that have been annotated with text serialization. They include examples of varying complexity including complex phosphopeptide spectra and a chimeric spectrum. The examples also include an annotated small molecule spectrum from MassBank,^40^ using features that are thus far available in the mzSpecLib and mzPAF basic support available for small molecules.

## Conclusion

We have presented here an overview of mzSpecLib, the new PSI spectral library format, and mzPAF, the new PSI peak annotation format. These data standards focus on the capability of encoding extensive metadata in a standardized mechanism. The standards attempt to be highly similar to what is already common practice (as seen in the commonly used MSP format) but with carefully documented and standardized specifications that have been debated and refined in an open community-based process.

These new standards also support features that were not available in previous formats, thereby providing standardization as well as additional functionality. We expect that these new standards will further accelerate the recent growth of spectral libraries in support of DIA, DDA, and SRM workflows by making high-quality reference libraries and reusable software components more broadly available. In the context of the FAIR (Findable, Accessible, Interoperable, Reusable) data principles,^41^ it is essential to have open formats for spectral libraries with the ability to trace library elements back to the original MS source files (the mass spectra), including published sources with USIs,^35^ analytes, InChI, SMILES, or protein accessions, enabling full transparency and traceability. The FAIR principles are further promoted when the spectral libraries provide the links to original public data sets with PXD identifiers and SDRF-Proteomics^42^ files that fully describe the samples and how the samples and data files are associated. Libraries should themselves be deposited and made publicly accessible in ProteomeXchange repositories with centrally managed identifiers for the libraries. Widespread adoption of the mzSpecLib and mzPAF standards in proteomics software would further accelerate the interoperability among those software tools.

There are many subfields of mass spectrometry, including cross-linking MS and glycoproteomics, that have special needs and potentially huge complexity both in the description of analytes as well as the fragment ion peaks. While we have produced a flexible set of formats that provide a basic foundation for supporting these types of data, practitioners in such subfields should come together if they are sufficiently motivated to produce best practice documents and examples that demonstrate how the many complexities in their spectra can be encoded via mzSpecLib and mzPAF in a common way.

PSI standards are developed via an open process in which all interested individuals and groups are encouraged to participate. Although data standards that are cooperatively developed inevitably take longer to complete, the resulting standards are more broadly applicable to many more use cases than those from independent initiatives. Broad participation is therefore essential for the successful generation of future standards for the proteomics community. See https://psidev.info/ for information about how to contribute to the PSI activities.
